# CO_2_ Adsorption Enhanced by Tuning the Layer Charge in a Clay Mineral

**DOI:** 10.1021/acs.langmuir.1c02467

**Published:** 2021-12-01

**Authors:** Kristoffer W. Bø Hunvik, Patrick Loch, Dirk Wallacher, Alexsandro Kirch, Leide P. Cavalcanti, Martin Rieß, Matthias Daab, Vegard Josvanger, Sven Grätz, Fabiano Yokaichiya, Kenneth Dahl Knudsen, Caetano Rodrigues Miranda, Josef Breu, Jon Otto Fossum

**Affiliations:** † Department of Physics, Norwegian University of Science and Technology, Høgskoleringen 5, 7491 Trondheim, Norway; ‡ Bavarian Polymer Institute and Department of Chemistry, University of Bayreuth, Universitätsstraße 30, D-95447 Bayreuth, Germany; § Helmholtz-Zentrum Berlin für Materialien und Energie, Hahn-Meitner-Platz 1, 14109 Berlin, Germany; ∥ Departamento de Física dos Materiais e Mecânica, Instituto de Física, Universidade de São Paulo, 05508-090 São Paulo, SP, Brazil; ⊥ Institute for Energy Technology (IFE), P.O. Box 40, N-2027 Kjeller, Norway; @ Inorganic Chemistry I, Ruhr-Universität Bochum, Universitätsstraße 150, 44780 Bochum, Germany

## Abstract

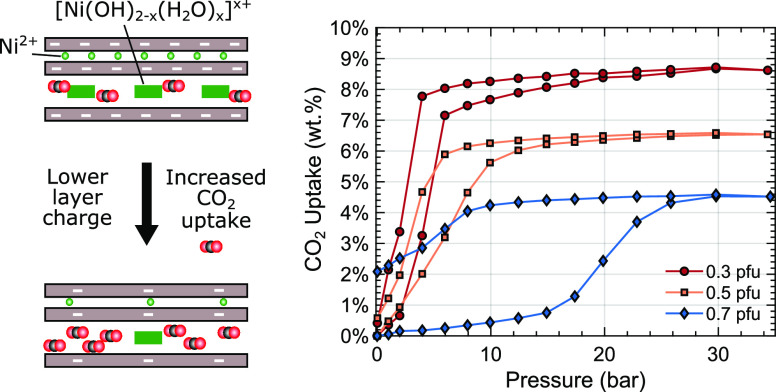

Due to the compact two-dimensional interlayer pore space and the high density of interlayer molecular adsorption sites, clay minerals are competitive adsorption materials for carbon dioxide capture. We demonstrate that with a decreasing interlayer surface charge in a clay mineral, the adsorption capacity for CO_2_ increases, while the pressure threshold for adsorption and swelling in response to CO_2_ decreases. Synthetic nickel-exchanged fluorohectorite was investigated with three different layer charges varying from 0.3 to 0.7 per formula unit of Si_4_O_10_F_2_. We associate the mechanism for the higher CO_2_ adsorption with more accessible space and adsorption sites for CO_2_ within the interlayers. The low onset pressure for the lower-charge clay is attributed to weaker cohesion due to the attractive electrostatic forces between the layers. The excess adsorption capacity of the clay is measured to be 8.6, 6.5, and 4.5 wt % for the lowest, intermediate, and highest layer charges, respectively. Upon release of CO_2_, the highest-layer charge clay retains significantly more CO_2_. This pressure hysteresis is related to the same cohesion mechanism, where CO_2_ is first released from the edges of the particles thereby closing exit paths and trapping the molecules in the center of the clay particles.

## Introduction

To mitigate greenhouse gas emissions, new technologies are required. As they are cheap, abundant, good CO_2_ adsorbers,^1−−15^ and present in cap-rock formations for carbon storage sites,^16^ smectite clay minerals are particularly important in this context. Understanding how carbon dioxide can selectively adsorb in the interlayers of clay minerals and tuning those mechanisms could both pave the way for new adsorbent materials and improve our understanding of the long-term stability of anthropogenic storage sites.

Smectite clay minerals are phyllosilicates consisting of 2:1 nanolayers built from two tetrahedral sheets sandwiching an octahedral sheet. Isomorphic substitutions in the tetrahedral or octahedral sheet cause a permanent charge of the 2:1 layer that is compensated by cations in the interlayer space. In dried smectite clay minerals, the two-dimensional interlayers are separated by ∼1 nm, providing ∼1000 km^2^ of accessible surface area per cubic meter of clay material. Within the interlayers there is a high density of molecular adsorption sites.^17^ The mechanism for molecular adsorption depends on the interlayer species at each adsorption site and their affinity for specific molecules. This means that the volumetric capacity for molecular adsorption in smectite clay minerals can be very high. In particular, this renders smectite clay minerals very competitive for CO_2_ capture, which was recently shown experimentally.^15^

Depending on their layer charge, these silicates can be divided into smectites, with a layer charge of 0.2–0.6 per formula unit (pfu) of Si_4_0_10_F_2_, and vermiculites, with a layer charge in the range of 0.6–0.9 pfu.^17^ The influence of the layer charge on the hydration properties of clay minerals has attracted considerable interest.^18−25^ Simulations on montmorillonite suggested that a higher layer charge facilitates the entry of water molecules into the clay interlayers and decreases CO_2_ adsorption, while a higher CO_2_/(CO_2_ + H_2_O) mole fraction is acquired when the charge is low.^26^ However, a systematic experimental study of how the layer charge influences CO_2_ adsorption in clay minerals has thus far not been conducted. Layer charge has been shown to have an effect on the adsorption of CH_4_ in hectorite,^27^ where a lower layer charge enhances CH_4_ adsorption.

In this work, we investigate the role of the layer charge in CO_2_ uptake and swelling in a synthetic clay mineral, fluorohectorite intercalated with nickel (Ni-Hec). Synthetic Hec shows a homogeneous charge density and, consequently, a uniform intracrystalline reactivity.^28^ It can be synthesized as large defect free particles,^29^ providing an excellent template for studying interactions with CO_2_ without the interference of defects or impurities. Ni-Hec has recently been shown to form a corrensite-like structure, with an ordered interstratifaction of chlorite-like layers with a composition of [Ni(OH)_0.83_(H_2_O)_1.17_]_0.37_^1.17+^, and hydrated smectite-like layers with Ni^2+^ cations.^30^ For this clay mineral, the hydration follows an atypical, partly continuous swelling behavior, which is related to the chlorite-like phase in the interlayer.^30,31^ The chlorite-like phase has also been demonstrated to be responsible for CO_2_ adsorption, where the molecules form a reversible bond with the intercalated nickel hydroxide.^14^ The CO_2_ could be grafted as a bicarbonate to the condensed nickel hydroxide species.^14^ To investigate the layer charge dependency in a systematic manner, we have conducted X-ray and neutron diffraction and gravimetric adsorption measurements on dry Ni-Hec prepared with three different layer charges, 0.3, 0.5, and 0.7 pfu. The samples were dried to at least 120 °C to ensure the removal of interlayer water and water coordinated to uncondensed interlayer cations, while preserving the clay layers and the nickel hydroxide in the chlorite-like layers.^30^ The experimental measurements are supported by first-principles calculations performed within the density functional theory (DFT) approach.

## Materials and Methods

### Materials

Na-fluorohectorite (Na-Hec) with a nominal composition of Na_*x*_(Mg_3–*x*_Li_*x*_)Si_4_O_10_F_2_ (*x* = 0.5 and 0.7) was prepared via melt synthesis according to published procedures.^29,32^ Therefore, the synthesis was carried out in gastight molybdenum crucibles. NaF (99.995%, Alfa Aesar), LiF (>99.9%, ChemPur), MgF_2_ (>99.9%, ChemPur), MgO (99.95%, Alfa Aesar), and SiO_2_ (Merck, fine granular quartz, purum) were mixed according to the nominal composition of Na-Hec. The crucible was heated to 1750 °C (15 °C/min), held at this temperature for 70 min, cooled to 1300 °C (55 °C/min) and then to 1050 °C (10 °C/min), and finally quenched by switching of the power. Subsequent long-term annealing was used to improve charge homogeneity and phase purity, resulting in phase pure and layer charge homogeneous materials. Na-fluorohectorite with *x* = 0.3 (Na-Hec_0.3_) was prepared after layer charge reduction of Na-Hec_0.5_ after repeated Mg exchange employing the Hofmann–Klemen effect.^33^ The layer charge was checked according to the literature.^34^ The cation exchange capacity (CEC) was determined using BaCl_2_ following DIN ISO 11260 and determined to be 75, 129, and 185 mequiv/100 g for layer charges of 0.3, 0.5, and 0.7 pfu, respectively. These results are in agreement with calculated values of 78, 130, and 194 mequiv/100 g, respectively. Ni-Hec was prepared by cation exchange of Na-Hec with a 0.2 M nickel acetate solution (>10-fold excess of the CEC, five times), following a published procedure.^30^ The exchanged Ni-Hec was washed five times with Millipore water.

Cation exchange of Ni-Hec with a long-chain *n*-alkylammonium solution (C16, C_16_H_33_NH_3_Cl) was performed with a 10-fold excess of CEC (80 °C, three times) to ensure complete exchange. The obtained C16-exchanged Ni-Hec was washed five times with ethanol/water (1:1) and once with ethanol (p.a.) and then dried at 80 °C. The composition of Ni-Hec_*x*_ (*x* = 0.3 and 0.7) was determined via inductively coupled plasma atomic emission spectroscopy (ICP-OES) according to Loch et al.,^30^ comparing the Ni content of the pristine and C16-exchanged Ni-Hec_*x*_. Approximately 20 mg of Ni-Hec_*x*_ equilibrated at a relative humidity of 43% was weighed into 15 mL clean Teflon flasks. C16-exchanged Ni-Hec_*x*_ was dried at 80 °C prior to the measurement. After addition of 1.5 mL of 30 wt % HCl (Merck), 0.5 mL of 85 wt % H_3_PO_4_ (Merck), 0.5 mL of 65 wt % HNO_3_ (Merck), and 1 mL of 48 wt % HBF_4_ (Merck), the sample was digested in a MLS 1200 Mega microwave digestion apparatus for 6.5 min and heated at 600 W (MLS GmbH, Mikrowellen-Labor-Systeme, Leutkirch, Germany). The closed sample container was cooled to room temperature, and the clear solution was diluted to 100 mL in a volumetric flask and analyzed on a PerkinElmer Avio 200 spectrometer.

### Powder X-ray Diffraction

Powder X-ray diffraction (PXRD) experiments during adsorption of CO_2_ were conducted at the KMC-2 beamline of the BESSY II light source at Helmholtz-Zentrum Berlin.^35^ A capillary-based sample cell system, centered in the synchrotron beam, was connected to a gas dosing system (Teledyne ISCO 260D). The temperature of the sample was directly measured by a K-type thermocouple mounted close to the measurement point and connected to a Eurotherm indicator unit 32h8i. A 0.01 mm wall thickness quartz capillary (Hilgenberg) with a diameter of 0.5 mm was glued in a ^1^/_4_ in. VCR weld gland (Swagelok). Before the measurements, the samples were filled in capillaries and dried for at least 2 h at 150 °C under high vacuum (10^–6^ mbar) in an *ex situ* dedicated capillary drying station. Subsequently, it was connected via a Swagelok thread to the gas handling system providing CO_2_ of quality N55 (99.9995%). All *in situ* measurements were carried out at 26 °C. The diffraction experiments were conducted in transmission geometry in the 2θ range of 2.5–14.5° using monochromatic synchrotron radiation (λ = 1.5406 Å). A full 360° rotation of the capillary in the beam was implemented by a script-controlled step motor. The diffraction images from the two-dimensional detector (Bruker Våntec 2000) were integrated using proprietary software. NIST silicon SRM 640a was used as the external standard for peak positions.

### Gravimetric Adsorption

CO_2_ adsorption measurements were conducted with an IsoSORP gravimetric sorption analyzer from Rubotherm. Each sample was prepared by being degassed at 120 ± 5 °C overnight under high vacuum. For each sample, three measurements were conducted with equilibration times of 1, 2, or 4 h at each pressure step from vacuum to 35 bar. The temperature was measured with a Pt-100 sensor placed directly underneath the sample crucible. It was surrounded by a double-walled thermostat controlled by a circulating water bath CC-K6 from Huber. The temperature stability of the sample was within 22.5 ± 1 °C. The suspension balance has a resolution of 0.01 mg and a reproducibility of <0.002% rdg (≈0.002 mg). The data for pressure, temperature, and sample weight were continuously recorded. The measured quantity is the excess adsorbed amount, which is obtained by correcting for the buoyancy of the skeletal volume of the sample material and the suspended metal parts (including the sample holder). The skeletal volume of the three samples was determined by individual helium isotherms. The buoyancy of the suspended metal parts was obtained by a blank measurement with CO_2_. The densities of helium and CO_2_ for the given pressure and temperature conditions were obtained from the equation of state data provided by NIST.^36^

### Computational Methodology

First-principles calculations based on the DFT were conducted to evaluate basal separation distances and adsorption energies. The simulations were performed using the Siesta package.^37^ The double-zeta plus polarization (DZP) and spin-polarized localized atomic orbital basis sets with a 400 Ry energy cutoff were used. The corrected generalized gradient approximation developed by Berland and Hyldgaard^38^ was employed to account for the van der Waals interactions. On the basis of the crystallographic structure of our previous work,^14^ the layer charges were modified by substituting Mg with Li atoms in the supercell (multiple unit cells). We systematically explored four layer charges (0.25, 0.5, 0.75, and 1.0 pfu), which were chosen to resemble the experimental stoichiometry. These charges were balanced with the interlayer Ni cations. For each stoichiometry, distinct Li and Ni interlayer atomic sites were fully considered and sampled for total energy minimization.

## Results and Discussion

### Structure Determination

Ni-Hec_0.5_ forms an ordered interstratification of a condensed [Ni(OH)_0.83_(H_2_O)_1.17_]_0.37_^1.17+^ species in one interlayer and hydrated Ni^2+^ cations in the adjacent layer.^30^ Due to the similar electron densities of the two interlayer species, the superstructure reflection is expected to be weak and was therefore amplified by enhancing the electron contrast of the interlayers. Cation exchange of the noncondensed Ni^2+^ interlayer cations with long-chain *n*-alkylammonium (C16, C_16_H_33_NH_3_Cl) led to a clearly visible superstructure as observed by PXRD.^30^ This procedure was used to identify a comparable condensed species and a possible superstructure in Ni-Hec_0.7_ and Ni-Hec_0.3_.

Similar to Ni-Hec_0.5_ (Figure 7 of ref (30)), C16-exchanged Ni-Hec_0.7_ shows a clearly visible reflection of an ordered interstratified superstructure (Figure S1). The basal spacing of 40.5 Å [coefficient of variation (CV) of 0.79] can be explained by the formation of an ordered interstratification of two strictly alternating interlayer species. The condensed nickel hydroxide species with a basal spacing of 14.5 Å and a paraffin-like arrangement of C16 cations with a basal spacing of 26 Å form a corrensite-like structure during the C16-exchange. The chemical composition was determined with ICP-OES according to Loch et al.,^30^ giving a composition of [[Ni(OH)_1.49_(H_2_O)_0.58_]_0.53_^0.58+^]_Int.1_ [[Ni(H_2_O)_6_]_0.38_^2+^]_Int.2_ [Mg_4.6_Li_1.4_]⟨Si_8_⟩O_20_F_4_ for Ni-Hec_0.7_.

Na-Hec_0.3_, which was used for the preparation of Ni-Hec_0.3_, cannot be obtained directly from melt synthesis. Thus, it requires a layer charge reduction procedure prior to cation exchange,^39^ which in turn lowers the layer charge homogeneity compared to those of Ni-Hec_0.5_ and Ni-Hec_0.7_. Therefore, C16-exchanged Ni-Hec_0.3_ showed no superstructure reflection (Figure S2). A basal spacing of 17.7 Å (CV = 0.42) is characteristic for a bilayer arrangement of C16^33,34,40^ in the interlayer space, indicating an occupation of C16 in every interlayer, and is in accordance with the layer charge of 0.3 pfu. The structure can consequently be seen as a random interstratification of condensed and noncondensed interlayer species with a composition of [[Ni(OH)_1.10_(H_2_O)_0.90_]_0.10_^0.90+^]_Int.1_ [[Ni(H_2_O)_6_]_0.25_^2+^]_Int.2_ [Mg_5.4_Li_0.6_]⟨Si_8_⟩O_20_F_4_.

### *In Situ* Powder X-ray Diffraction

**Figure 1:**
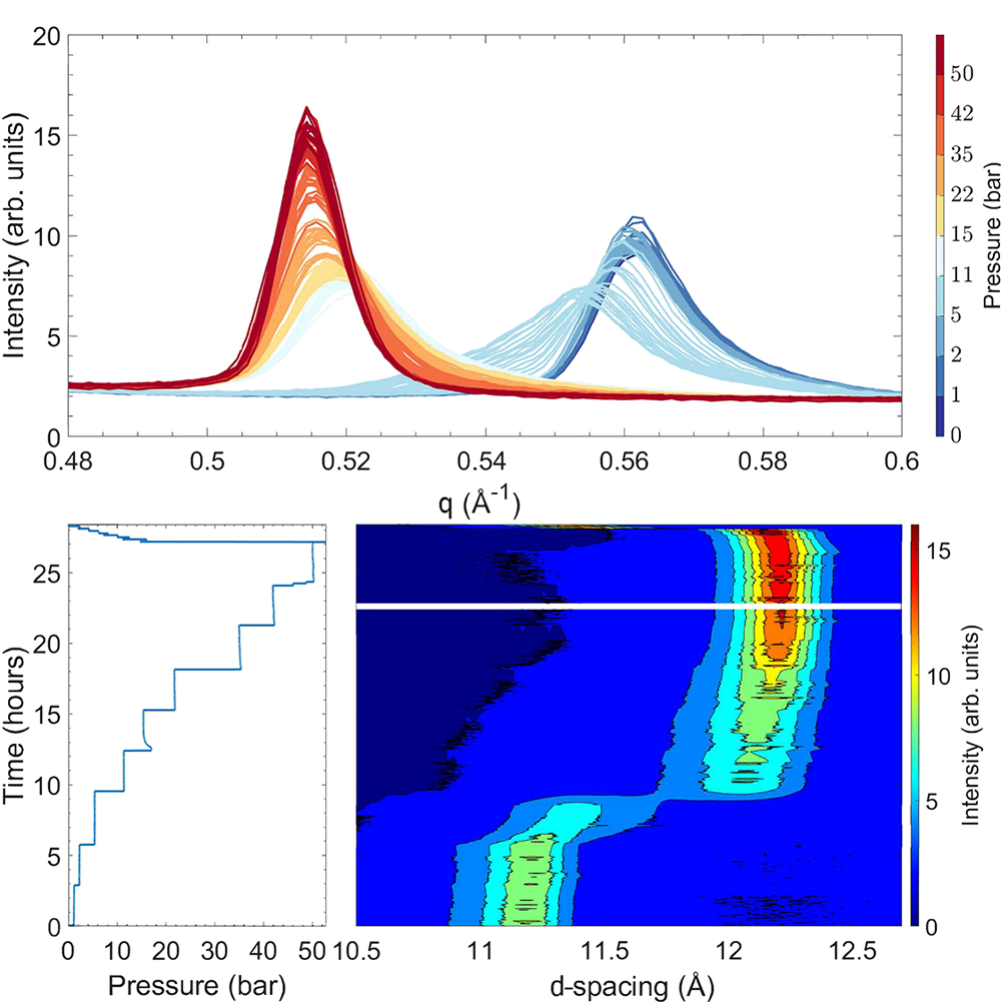
Evolution of the (001) Bragg reflection as a function of pressure at 26 °C for Ni-Hec_0.3_ recorded via PXRD. The bottom left panel shows the measured pressure of CO_2_ applied on the sample as a function of time, and the bottom right panel shows a contour plot in which the horizontal axis corresponds to the *d* spacing (*d* = 2π/*q*). The vertical axis corresponds to time scaled by the same horizontal axis as for the pressure, and the color gradient represents the intensity. In the top panel, the evolution of the (001) Bragg reflection is plotted with colors indicating the approximate pressure.

**Figure 2:**
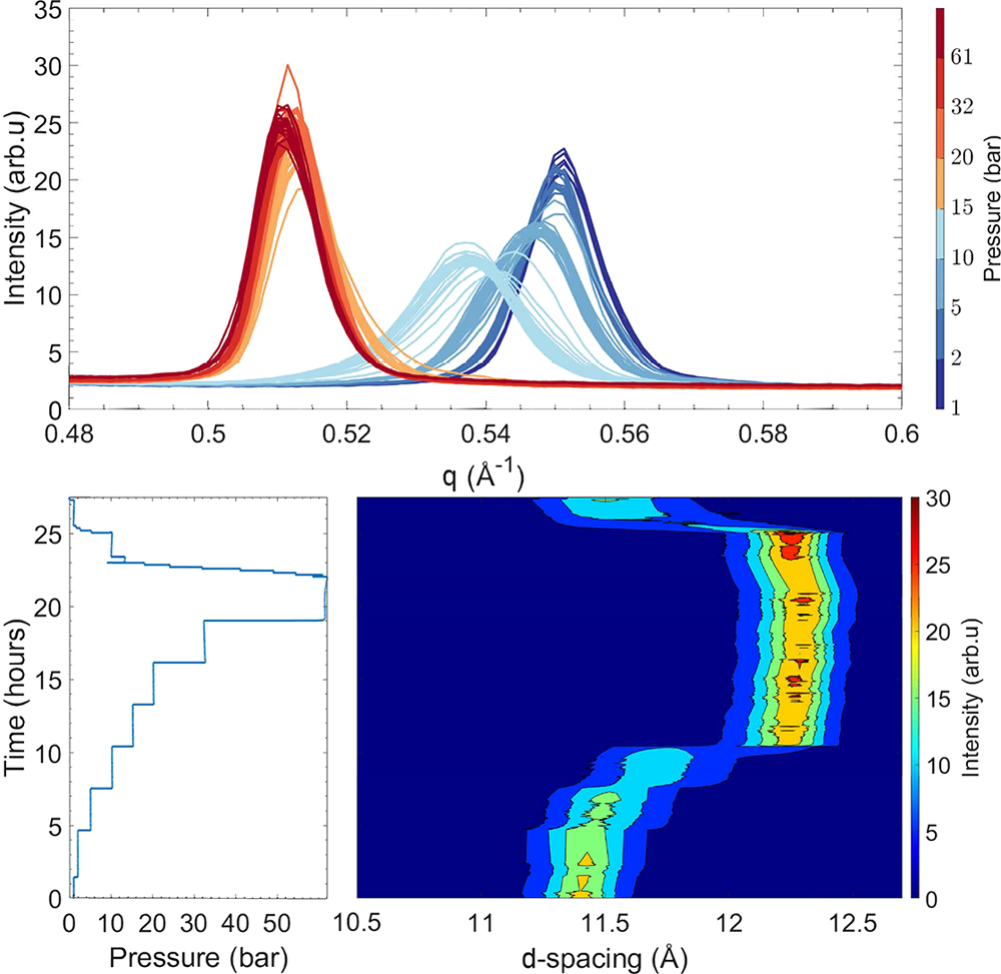
Evolution of the (002) Bragg reflection as a function of pressure at 26 °C for Ni-Hec_0.5_ recorded via PXRD. The bottom left panel shows the measured pressure of CO_2_ applied on the sample as a function of time, and the bottom right panel shows a contour plot in which the horizontal axis corresponds to *d* spacing (*d* = 2π/*q*). The vertical axis corresponds to time scaled by the same horizontal axis as for the pressure, and the color gradient represents the intensity. In the top panel, the evolution of the (002) Bragg reflection is plotted with colors indicating the approximate pressure.

**Figure 3:**
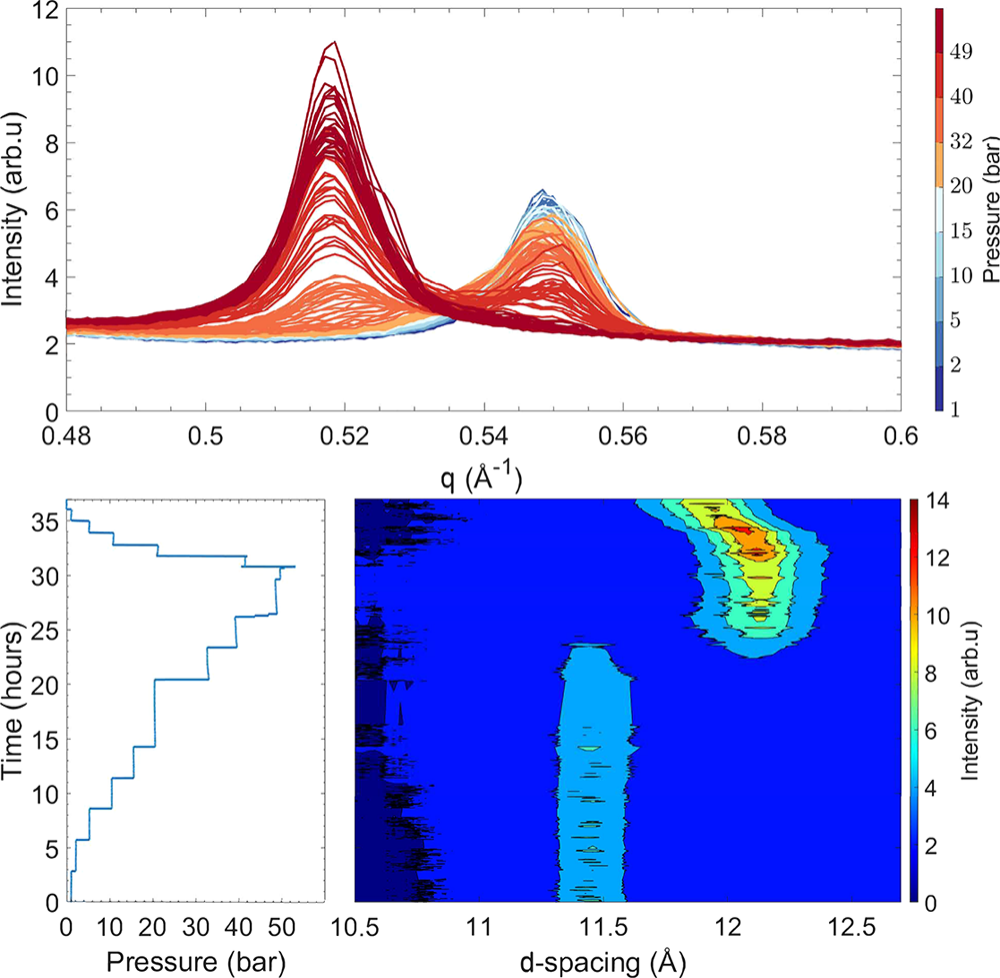
Evolution of the (002) Bragg reflection as a function of pressure at 26 °C for Ni-Hec_0.7_ recorded via PXRD. The bottom left panel shows the measured pressure of CO_2_ applied on the sample as a function of time, and the bottom right panel shows a contour plot in which the horizontal axis corresponds to the *d* spacing (*d* = 2π/*q*). The vertical axis corresponds to time scaled by the same horizontal axis as for the pressure, and the color gradient represents the intensity. In the top panel, the evolution of (002) Bragg reflection is plotted with colors indicating the approximate pressure.

The evolution of the (001) or (002) Bragg reflection upon CO_2_ exposure for Ni-Hec_*x*_ dried at 150 °C with a layer charge of 0.3, 0.5, and 0.7 pfu is shown in Figures 1–3. As no ordered superstructure is observed for Ni-Hec_0.3_ after C16 exchange, these results represent the (001) Bragg reflection of a random interstratification (Figure 1), while for Ni-Hec_0.5_ and Ni-Hec_0.7_, it corresponds to the (002) reflection of an ordered interstratification (Figures 2 and 3). Similar to what was observed for Ni-Hec_0.5_ by Hunvik et al.,^14^ with an increasing pressure of CO_2_ the Bragg reflection moves to lower *q* values as a result of the swelling process. Swelling is an inherent one-dimensional process, in which the Bragg reflections gradually move as the number of swollen interlayers increases and thus the relative contribution of the interstratified stacks becomes larger.

For dried Ni-Hec_0.3_, initially an asymmetric (001) Bragg reflection at 0.563 Å^–1^ (*d*_001_ = 11.15 Å) under vacuum (0.07 bar) is observed (Figure 1). During the CO_2_ exposure, no changes were observed at 1 and 2 bar of CO_2_. As the pressure is increased to 5 bar, the peak gradually shifts, and the sample starts to swell. At 11 bar, the intensity is shifted from the educt to the product phase, slowly becoming more symmetric toward the final pressure. At the final pressure of 50 bar of CO_2_, a symmetric (001) Bragg reflection at 0.514 Å^–1^ (*d*_001_ = 12.21 Å) is observed. The pressure was released quickly after CO_2_ exposure, and a symmetric peak appears, centered at 0.556 Å^–1^ (*d*_001_ = 11.30 Å) (Figure S3). The symmetric peak indicates a higher-order stacked structure, while the value of the basal spacing reveals the release of most of the CO_2_.

For dried Ni-Hec_0.5_, initially a symmetric peak at 0.551 Å^–1^ (*d*_002_ = 11.39 Å) is observed, corresponding to the (002) reflection of the ordered interstratified structure (Figure 2). Similar to the observation of Hunvik et al.,^14^ up to 10 bar the shifts are marginal for each pressure step. At 10 bar, the reflection gradually shifts toward the product phase, ending up at 15 bar as a symmetric peak with only minor changes, before reaching the final pressure. At the final pressure of 60 bar, a symmetric peak at 0.512 Å^–1^ (*d*_002_ = 12.26 Å) is observed. When the the pressure is decreased, CO_2_ is released and the reflection shifts back to 0.547 Å^–1^ (*d*_002_ = 11.48 Å), forming a significantly broader peak at higher *d* spacings compared to that of the initial state. Compared to Ni-Hec_0.3_, the onset of swelling is located at a higher pressure, while the end state after pressure is decreased is less ordered, as judged from the peak broadening. This is likely a result of some interlayers still retaining CO_2_ (discussed below) and suggests that the cohesion of the layers in this case is stronger than that in Ni-Hec_0.3_, preventing the complete desorption of CO_2_. Included in the Supporting Information are neutron diffraction measurements (Figures S4 and S5), where the sample was given more time to equilibrate. Consequently, it is clear that Ni-Hec_0.5_ returns to its initial state given enough time under vacuum (0.003 bar), which confirms the influence of the higher layer charge on CO_2_ adsorption.

For dried Ni-Hec_0.7_, a symmetric (002) Bragg reflection of the corrensite structure at 0.548 Å^–1^ (*d*_002_ = 11.45 Å) is observed (Figure 3). During the exposure to CO_2_, the peak shape remains almost unchanged until the pressure reaches 32 bar, when the level of the product phase starts to increase at the expense of the educt phase. At the final pressure of 50 bar, a symmetric peak at 0.518 Å^–1^ (*d*_002_ = 12.11 Å) is observed. When the pressure is subsequently reduced, the (002) Bragg reflection slowly returns to higher *q* values, corresponding to a decrease in the basal spacing. However, the sample does not reach its initial state and a broad peak centered at 0.527 Å^–1^ (*d*_002_ = 11.90 Å) is observed, which is even broader than in Ni-Hec_0.5_. Again, swelling and shrinking in response to CO_2_ result in two well-defined states, where the onset is at an even higher pressure than for the two previous samples with lower layer charges (0.3 and 0.5 pfu).

For the X-ray diffraction experiments, the equilibration time was shorter than for the neutron diffraction experiments (Figure S6). In the latter case, when the pressure is decreased, the basal spacing of Ni-Hec_0.7_ does not return to its initial state, indicating a certain retention of CO_2_ even after longer equilibration times at low pressure. Consequently, the higher layer charge also assists in the retention of CO_2_ and confirms the influence of the layer charge on the swelling process.

**Figure 4:**
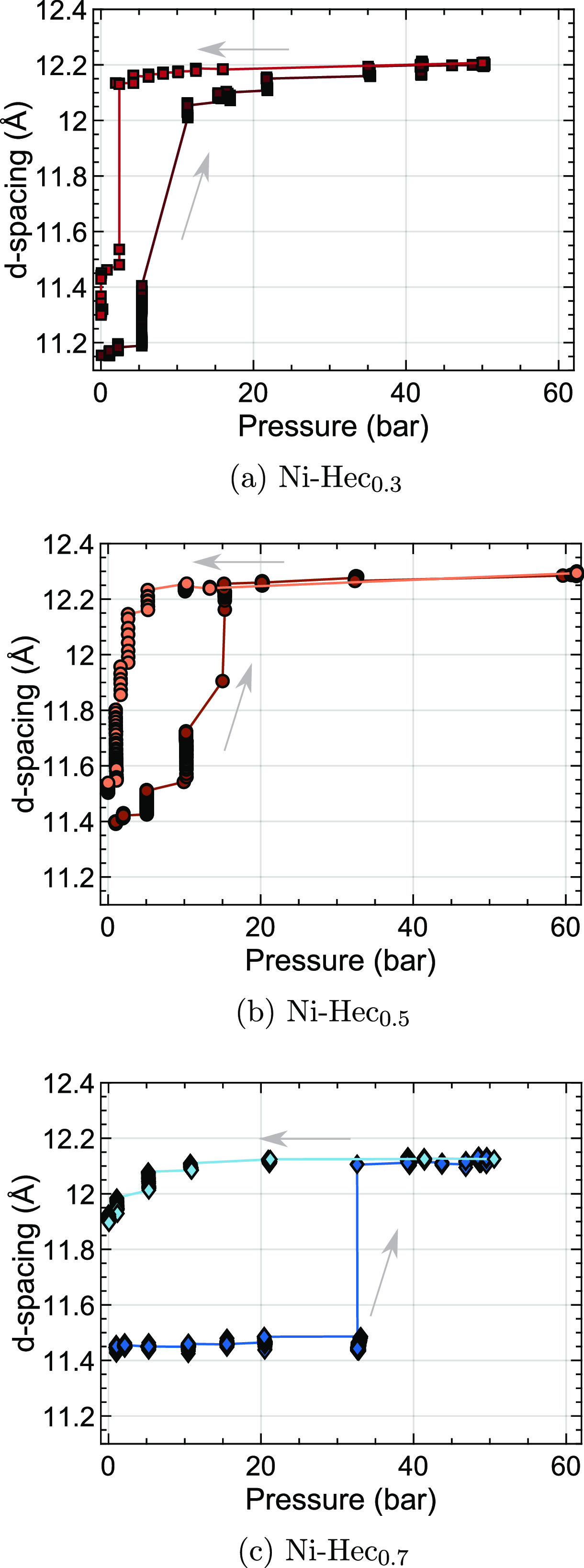
Evolution of the most prominent feature of the (001)/(002) Bragg reflection as a function of pressure at 26 °C for Ni-Hec_0.3_, Ni-Hec_0.5_, and Ni-Hec_0.7_. The arrows show the swelling hysteresis with the increase or decrease in pressure.

The three samples are compared in Figure 4, where the *d* spacing of the most intense peak of the (001)/(002) Bragg reflection as a function of pressure is shown. It is evident from the discussion above and Figure 4 that Ni-Hec with a higher layer charge shows a higher onset pressure for swelling upon CO_2_ exposure and, concomitantly, greater retention of the CO_2_. This results in a larger hysteresis loop starting from Ni-Hec_0.3_ to Ni-Hec_0.7_, where Ni-Hec_0.7_ clearly resides in a partly swollen state even after the CO_2_ pressure is decreased. The slightly higher basal spacings of Ni-Hec_0.3_ and Ni-Hec_0.5_ might indicate a certain retention of CO_2_ but are negligible compared to that of Ni-Hec_0.7_ and can be due to a higher stacking order or differences in the degree of condensation of the chlorite-like layers.

### Gravimetric Adsorption

**Figure 5:**
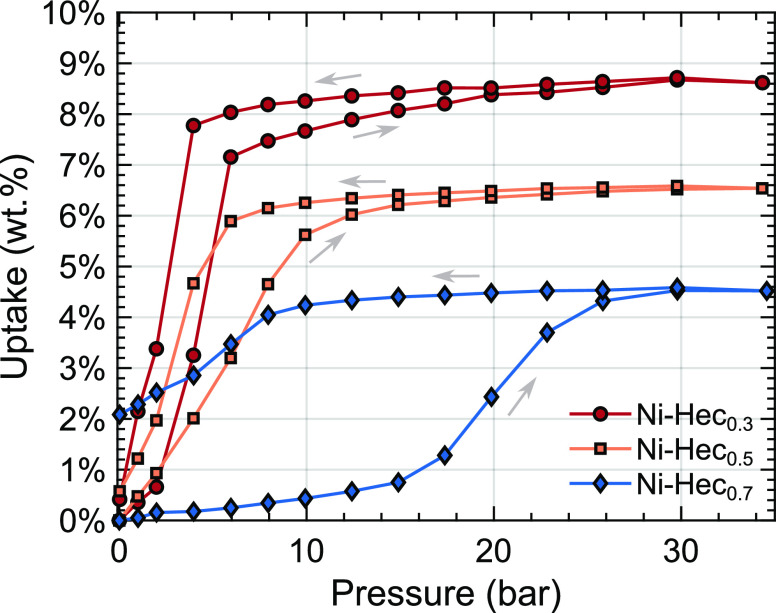
Comparison of the gravimetric adsorption capacity in dried Ni-Hec with layer charges Ni-Hec_0.3_, Ni-Hec_0.5_, and Ni-Hec_0.7_ equilibrated for 4 h at each pressure step. The arrows show the sorption hysteresis with the increase or decrease in pressure.

To quantify CO_2_ uptake as a function of the layer charge of Ni-Hec, gravimetric adsorption measurements were performed. The gravimetric excess sorption of Ni-Hec with the three different layer charges is shown in Figure 5. The respective densities obtained from an He isotherm are listed in Table S1. In agreement with the PXRD results, the adsorption should largely take place in the interlayer space, as the N_2_ physisorption isotherm for Ni-Hec_0.5_ (Figure S7) shows only limited adsorption and no significant BET surface area (1.8 m^2^/g). Included in Figure S8 are gravimetric data where the equilibration time at each pressure step is 1, 2, and 4 h. We observed that the equilibration time has no effect on Ni-Hec_0.3_. For Ni-Hec_0.5_ and Ni-Hec_0.7_, a dependency on equilibration is noticed in the decrease in the size of the hysteresis loop with an increase in the equilibration time. However, for all samples, the final uptake is unaffected by exposure to CO_2_ with these equilibration times. The additional buoyancy due to the increase in volume by the clay swelling in response to CO_2_ is not included in the data presented in Figure 5. At the final pressure with a fully swollen state, this will add an additional 3–4% to the final excess adsorption capacity.

For Ni-Hec_0.3_ (Figure 5), the uptake of CO_2_ starts immediately between 2 and 6 bar. The uptake rises only slightly above 6 bar, until the curve flattens around 20 bar, achieving a maximum uptake of 8.6 wt % (2.14 mmol/g) at 35 bar. Upon desorption, no significant mass loss was observed until approximately 4 bar. Below that point, a large step occurred and most of the CO_2_ is released when the lowest pressure (0.02 bar) is reached. For Ni-Hec_0.5_ (Figure 5), the uptake starts immediately similar to Ni-Hec_0.3_ and follows a continuous trend up to ∼12 bar, where the curve starts to flatten out. At 35 bar, a maximum excess adsorption of 6.5 wt % (1.59 mmol/g) was achieved, lower than the uptake observed for Ni-Hec_0.3_. Upon desorption, only a small mass loss until approximately 8 bar can be observed, after which most of the CO_2_ is released at the lowest pressure (0.01 bar) and approximately 0.5 wt % CO_2_ remained in the sample. For Ni-Hec_0.7_ (Figure 5), only a minor uptake before 13 bar is found, which significantly increases until 30 bar after which the curve flattens. At 35 bar, a maximum excess adsorption of 4.5 wt %, 1.08 mmol/g, is observed. Upon desorption, the sample does not release any significant amount of CO_2_ above 12 bar. Ultimately, between 2 and 2.5 wt % of CO_2_ was retained in the sample.

The shape of the adsorption isotherms resembles that of the water adsorption isotherms for Na-Hec,^28,41^ where the adsorption occurs between two discrete states. Due to the limited adsorption of Ni-Hec_0.7_ before the pressure threshold, any significant adsorption of CO_2_ in the bulk can be ruled out, which would otherwise produce a more gradual adsorption isotherm. This is consistent with the swelling observed by *in situ* PXRD that occurs only between two defined states.

In terms of uptake, layer charge plays a distinct role, with a higher uptake for a lower layer charge. At 35 bar, the CO_2_ uptake values are 8.6, 6.5, and 4.5 wt % for Ni-Hec with layer charges of 0.3, 0.5, and 0.7 pfu, respectively. Previously, we demonstrated that the adsorption mechanism in Ni-Hec_0.5_ is controlled by an interaction with the [Ni(OH)_0.83_(H_2_O)_1.17_]_0.37_^1.17+^ species in the interlayer,^14^ and the Ni^2+^ cations in the smectite-like layers are unresponsive to CO_2_ under anhydrous conditions. For the low-charge clay, less interlayer species is required to counterbalance the layer charge, and therefore, more space is available in the interlayer for CO_2_. This is also confirmed by the measured lower density from the He isotherm (Table S1) for the lower-charge clay, because greater formation of nickel hydroxide would result in a higher density. Given the low density of [[Ni(OH)_1.10_(H_2_O)_0.90_]_0.10_^0.90+^] species for Ni-Hec_0.3_ some of the CO_2_ may be attached to the interlayer species, and some may be occupying the interlayer space available between the condensed islands. Previous observations of CH_4_ adsorption in hectorite with varying layer charges find greater CH_4_ adsorption for a lower layer charge.^7^ In accordance with our conclusions, this was related to more pore space being available due to fewer cations being required in the interlayer to balance the lower layer charge.

**Figure 6:**
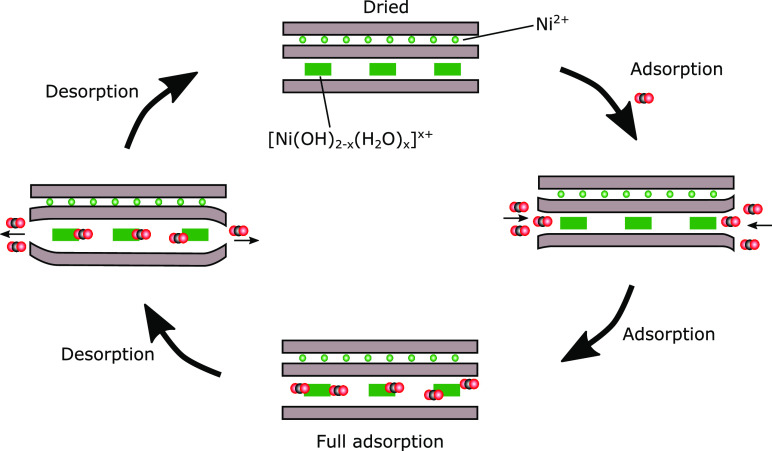
Sketch of the CO_2_ adsorption process in clay. The CO_2_ penetrates the clay from the edges of the clay platelet, slowly expanding the clay as the CO_2_ enters. At full adsorption, the clay is fully expanded. As the pressure is decreased, the CO_2_ molecules close to the edges are the first to leave the clay, forming a pocket inside with trapped CO_2_. With a decrease in the pressure and given enough time, the clay will slowly return to its initial dry and collapsed state.

The hysteresis behavior is also influenced by the layer charge. Ni-Hec_0.7_ (Figure 5) starts adsorbing at much higher pressures than the two other samples. A difference is also apparent in that for the lower-charge samples (Ni-Hec_0.3_ and Ni-Hec_0.5_) almost all of the CO_2_ can be released, whereas in the case of the high layer charge (Ni-Hec_0.7_), CO_2_ remains in the sample under the used conditions. A higher layer charge results in stronger electrostatic cohesion between the clay layers, creating a higher energy barrier for the CO_2_ to enter the interlayer. A possible mechanism is illustrated in Figure 6, showing swelling that first occurs from the edges of the clay particle. Upon desorption, the CO_2_ first leaves from regions near the edges. This could partially close the exit paths, trapping some of the CO_2_ by the electrostatic cohesion of the layers. This effect would be more important for a higher layer charge and, consequently, result in the greater retention we observe. This is similar to the so-called “ring mechanism” proposed by Weiss et al.^42^ for kaolinite intercalation, which was also observed for Hec by Stöcker et al.^43^ Due to the large size of the tactoid, intercalation occurs along all edges of the particle instead of via a “one-sided wedge” mechanism.

These results show a larger dependency on layer charge for CO_2_ adsorption and swelling compared to what has been observed for H_2_O adsorption and/or swelling.^18,19^ The crystalline swelling upon hydration of clays depends on the Coulombic and van der Waals attraction, hydration and Born repulsion and is inherently hysteretic.^44^ For synthetic saponites, where the charge is located in the tetrahedral sheet, swelling upon exposure to water occurs at lower values of relative humidity as the layer charge increases.^18^ In addition, the increase in layer charge led to an increasing amount of water adsorbed in this clay mineral, due to the larger amount of interlayer cations allowing greater hydration.^18^ For hectorite, on the contrary, with an origin of the layer charge in the octahedral sheet, the water adsorption isotherms and the amount of water adsorbed were independent of the location and the amount of layer charge.^19^ Water strongly interacts with the interlayer cation, an interaction that is significantly stronger than the Coulomb attraction between the layers and the interlayer cation. For CO_2_, there is a weaker interaction with the interlayer species than for water. The resulting layer–layer repulsion force from the presence of CO_2_ could then be comparable to the Coulomb attraction, which could explain the large dependency of layer charge for CO_2_ swelling as opposed to the H_2_O case.

### DFT Calculations

**Figure 7:**
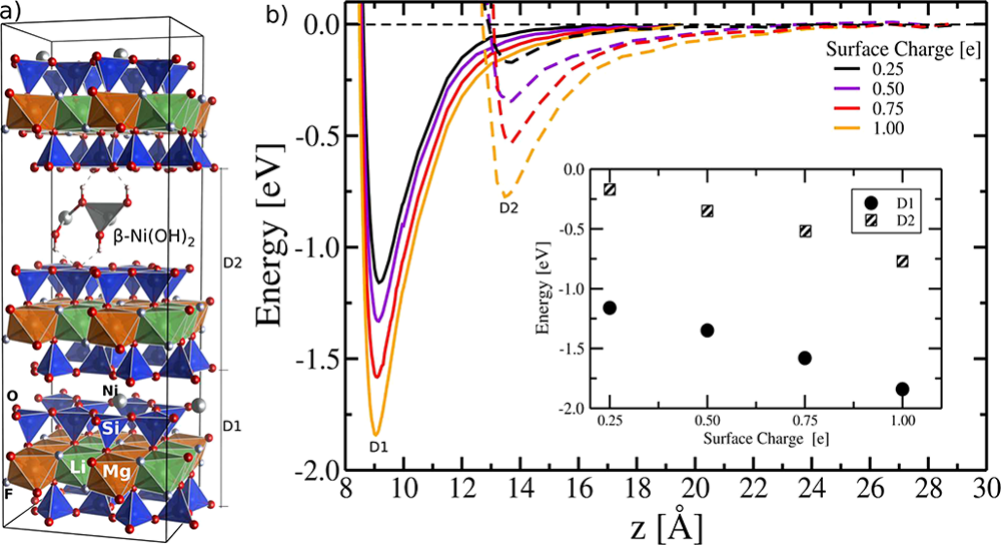
(a) Side view of the Hec molecular model highlighting the atomic configuration with a layer charge of 1.0 pfu (unit cell area of 9.2 Å × 5.3 Å) achieved by the substitution of two Mg atoms with Li atoms. (b) Energy curves for the dehydrated smectite (*D*_1_) and for the β-Ni(OH)_2_ island (*D*_2_) in the interlayer space considering the role of the surface charges. The equilibrium position indicates the basal separation distance, while the depth of the potential well may be interpreted as the cohesion energy per unit cell. The basal *d* spacing can be obtained by the term (*D*_1_ + *D*_2_)/2. The inset shows the energy minimum as a function of surface charge.

This interpretation is reinforced by DFT simulations of the system. The cohesion energy of the layers significantly increases with layer charge (Figure 7). For the chlorite-like layer where the CO_2_ is believed to enter, the value of the energy minimum more than doubles. In addition, basal separation distances were determined as a function of the surface charge. We observed that the equilibrium configurations of dried smectites have a minor dependence (<0.1 Å) on the surface charge, which is in agreement with previous studies.^26^

### Comparison to Other Systems

**Figure 8:**
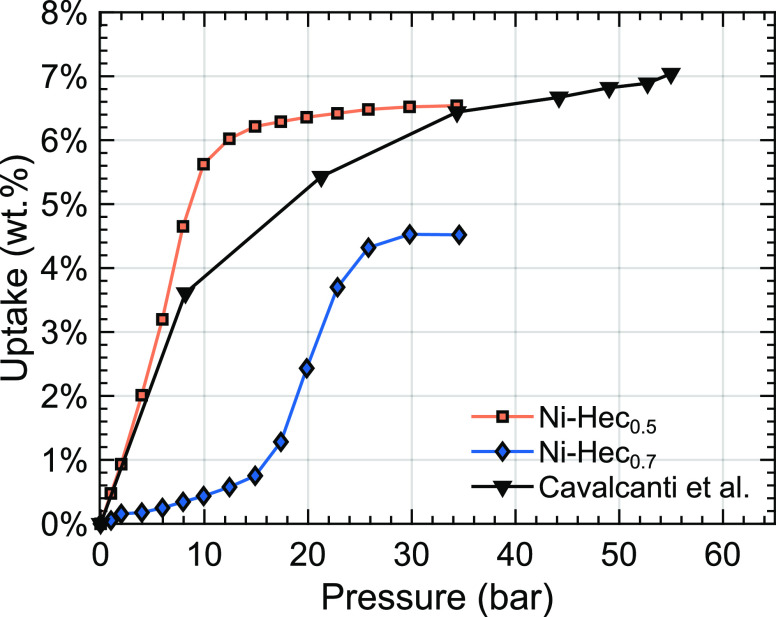
Comparison of the adsorption capacity of Ni-Hec_0.5_ and Ni-Hec_0.7_ compared with results on a Ni-Hec cation-exchanged from a commercial Corning sample.^15^

To compare our results with similar measurements on a commercial Ni-exchanged Hec from Corning by Cavalcanti et al.,^15^ where the bulk density (≈0.7 g/cm^3^) of the samples was used to estimate the effective volume, we have to use the density acquired from an He isotherm. The final uptake in Cavalcanti et al.^15^ is readjusted to 7 wt %, by assuming that the density is the same as that of our current Ni-Hec_0.7_. The uptake behavior for this sample is in accordance with our observed layer charge dependence, as shown in Figure 8, because the sample of Cavalcanti et al.^15^ has a layer charge of ∼0.6 pfu on average. Still, a direct comparison is difficult as the samples are different in terms of origin, preparation, layer charge, and phase purity. Compared to other clay minerals measured under similar conditions, like dried SWy-2 and STx-1 where a maximum excess adsorption of 0.250–0.650 mmol/g under high pressure has been reported,^4,9−11^ our results show a higher adsorption capacity. Compared to the capacity of amine-modified montmorillonite (2.4 mmol/g),^45^ our reported capacity is a little lower. However, because both our current and previous^14^ investigations indicate that only half of the interlayers are active in the adsorption process, there is a potential to double the capacity if [Ni(OH)_2–*x*_(H_2_O)_*x*_]_*y*_^*x*+^ islands can be condensed in all interlayers.

## Conclusion

Our findings demonstrate that the uptake of CO_2_ in the interlayer of a clay mineral increases with a decrease in layer charge. We suggest that this is due to more accessible space and adsorption sites for CO_2_ within the interlayers. Moreover, the uptake pressure threshold decreases with a lower layer charge, which we explain by weaker cohesion between the layers.

In future work, these mechanisms will be examined for natural clay minerals, such as montmorillonite. Performing the cation exchange at a slightly increased pH (>8) is shown in the literature^46−48^ to produce condensed chlorite-type interlayers only and therefore may double the adsorption capacity for CO_2_. Given the compact nature of clay minerals, this can make them volumetrically competitive compared to MOFs and mesoporous carbon.^15^ The mechanism for CO_2_ capture by clay minerals presented here will also be investigated for a series of transition metals known for their condensation tendency.

The optimum layer charge for Ni-Hec coincides with the layer charge for natural montmorillonite, which is the main ingredient in commercial bentonite. Therefore, the impact of our results could be important for the implementation of clay minerals for industrial carbon capture, separation, and sequestration processes. In addition, as clay minerals are present in cap-rock formations for anthropogenic storage sites, these results provide new knowledge that could be relevant for the long-term stability of such reservoirs.

## Supporting Information Available

Supporting neutron diffraction, gravimetric adsorption, and X-ray diffraction measurements, sample densities, and a BET isotherm (PDF)
