# Supplementary material for: CO2 Adsorption Enhanced by Tuning the Layer Charge in a Clay Mineral
Source: Langmuir. 2021 Dec 1;37(49):14491–9. doi: 10.1021/acs.langmuir.1c02467 (PMC8675214; doi:10.1021/acs.langmuir.1c02467)
Supplement: Supplementary file 1 — la1c02467_si_001.pdf [file la1c02467_si_001.pdf]

Supporting Information: CO_2 Adsorption Enhanced by Tuning the Layer Charge in a Clay Mineral

Kristoffer W. Bø Hunvik,[*,†] Patrick Loch,[‡] Dirk Wallacher,[¶] Alexsandro Kirch,[§] Leide P. Cavalcanti,[‖,#] Martin Rieß,[‡] Matthias Daab,[‡] Vegard Josvanger,[†] Sven Grätz,[⊥] Fabiano Yokaichiya,[¶] Kenneth Dahl Knudsen,[‖,†] Caetano Rodrigues Miranda,[§] Josef Breu,[‡] and Jon Otto Fossum[*,†]

†*Department of Physics, Norwegian University of Science and Technology, Høgskoleringen 5, 7491 Trondheim, Norway*

‡*Bavarian Polymer Institute and Department of Chemistry, University of Bayreuth, Universitätsstraße 30, D-95447 Bayreuth, Germany*

¶*Helmholtz-Zentrum Berlin für Materialien und Energie, Hahn-Meitner-Platz 1 14109, Berlin, Germany*

§*Departamento de Física dos Materiais e Mecânica, Instituto de Física, Universidade de São Paulo, 05508-090 São Paulo, SP, Brasil*

‖*Institute for Energy Technology (IFE), P.O. Box 40, N-2027 Kjeller, Norway*

⊥*Inorganic Chemistry I, Ruhr-Universität Bochum, Universitätsstraße 150, 44780 Bochum, Germany*

#*Current address: ISIS Neutron and Muon Source, STFC Rutherford Appleton Laboratory, Chilton OX11 0QX, Didcot, U.K.*

E-mail: kristoffer.hunvik@ntnu.no; jon.fossum@ntnu.no

X-ray diffraction of C16-exchanged Ni-Hec

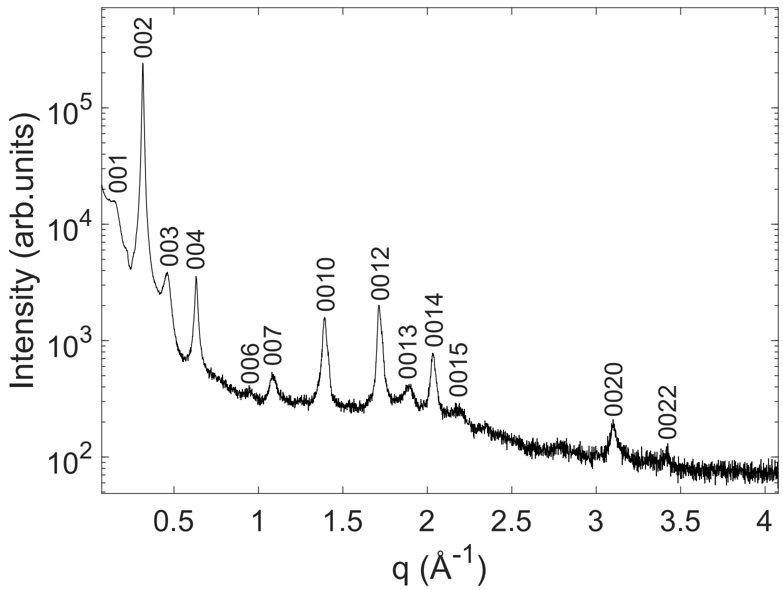

Figure S1: Powder X-ray diffraction pattern of Ni-Hec$_{0.7}$ exchanged with C16 ($C_{16}H_{33}NH_3Cl$).

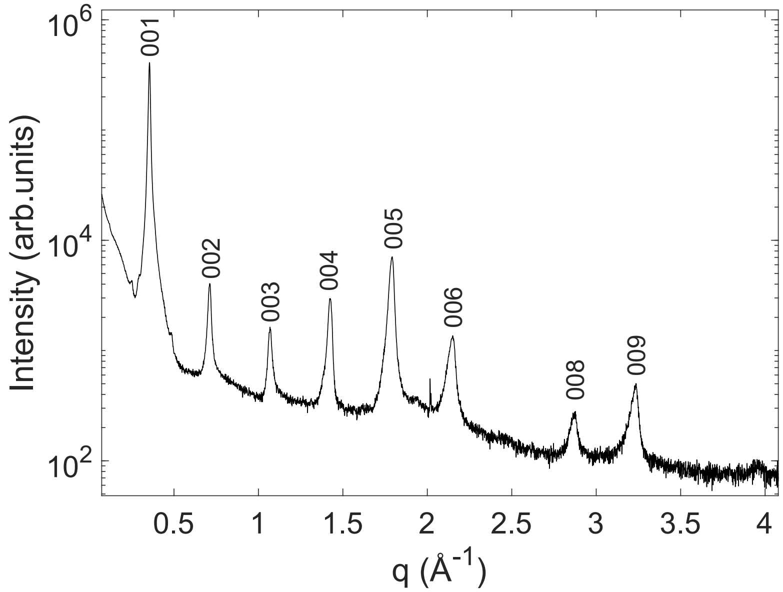

Figure S2: Powder X-ray diffraction pattern of Ni-Hec$_{0.3}$ exchanged with C16 ($C_{16}H_{33}NH_3Cl$).

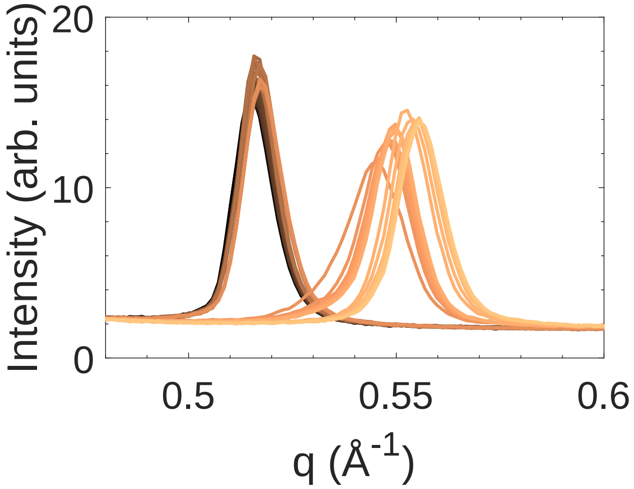

Figure S3: The evolution of the (001) Bragg reflection as a upon reducing CO_2 pressure at 26 °C for Ni-Hec$_{0.3}$ measured by X-ray diffraction. The copper colorgradient indicates the pressure reduction and time, where the measurements at lowest pressure are in the brightest color.

Neutron Diffraction

Neutron diffraction measurements were carried out at the flat-cone diffractometer E2[S1] at the BER II reactor of Helmholtz-Zentrum Berlin. The instrument utilized a Debye-Scherrer geometry with wavelength $\lambda = 2.4$ Å. The powdered samples were first dried overnight *ex situ* at 150 °C under high vacuum, and then completely filled in 0.5 mL aluminium containers in inert nitrogen atmosphere in a glovebox. The sample holder was connected to a gas dosing system (Teledyne ISCO 260D). The temperature was controlled by a pulse tube based temperature environment at 26 °C. The pressure was increased in steps from high vacuum to 50 bar at constant temperature, and continuous 5 minute measurements were conducted in the measurement range 3.9 ° $< 2\theta <$ 79.3 °.

Figure S4 shows the evolution of the (002) Bragg reflection as a function of pressure of Ni-Hec$_{0.5}$. The pressure was increased and decreased step wise using approximately 2 hour at each step, both for increasing and decreasing pressure. The evolution of the peak with the highest intensity at each pressure step is shown in Figure S5, and the hysteresis is quite similar to what is observed for Ni-Hec$_{0.5}$ by X-rays in Figure 4. Here it is clearly observed

that Ni-Hec$_{0.5}$ returns to it's initial state given enough time under vacuum (0.003 bar). Note that the samples were prepared from two different batches, resulting in a slight difference between the initial and final position of the (002) Bragg reflection for sample measured by neutrons and X-rays respectively. The condensation of the corrensite structure[S2,S3] is sensitive to the preparation protocol, and could result in a different amount/distribution between chlorite-like and smectite-like layers. The peak with the highest intensity at each pressure step is given in Figure S6 for Ni-Hec$_{0.7}$, showing a similar behavior to that observed in Figure 4 (main text) by X-rays. The sample swells in the response to CO_2, reaching its full swollen state at 35 bar. When reducing the pressure Ni-Hec$_{0.7}$ does not return to its initial state, indicating some retention of CO_2 even under longer equilibration times.

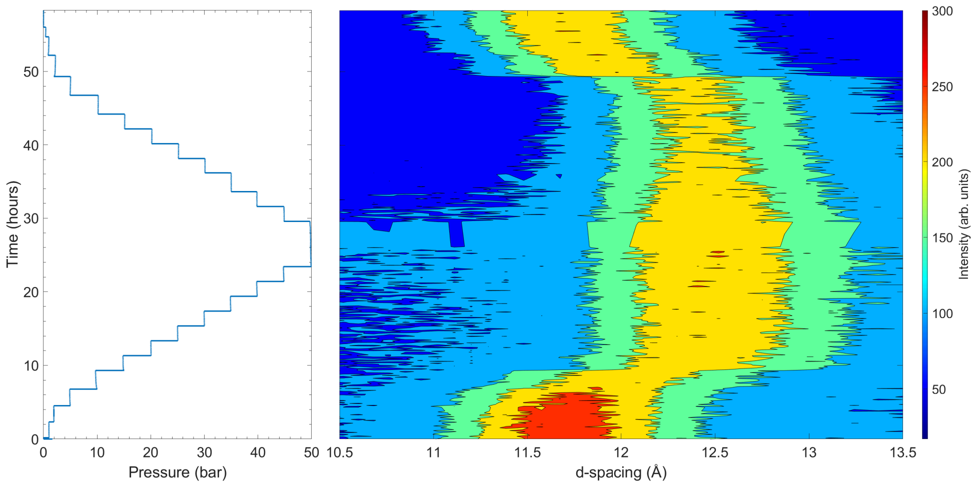

Figure S4: Evolution of the (002) Bragg reflection as a function of pressure at 26 °C for Ni-Hec$_{0.5}$. The left panel is the measured pressure of CO_2 applied on the sample as function of time, the right panel is a contour plot where the horizontal axis corresponds to d-spacing ($d = 2\pi/q$), the vertical axis corresponds to time scaled by the same horizontal axis as for the pressure and the colorgradient represents the intensity.

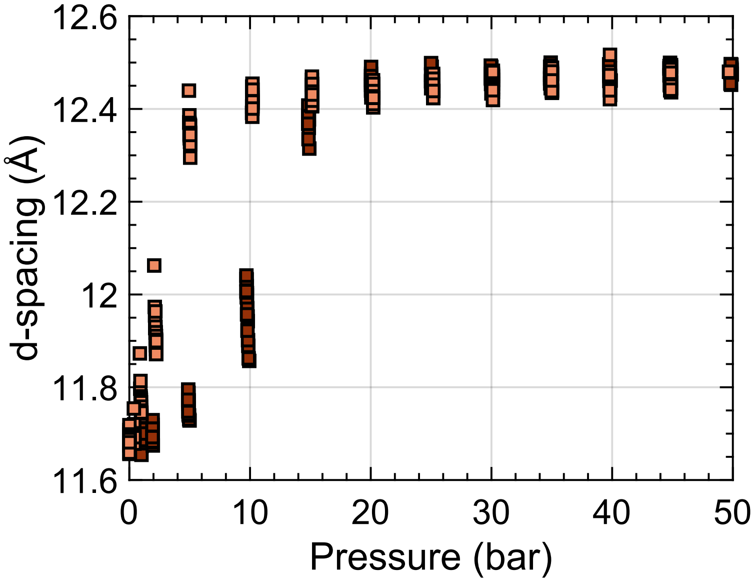

Figure S5: Evolution of the most prominent feature of the (002) Bragg reflection as a function of pressure at 26 °C for Ni-Hec$_{0.5}$ recorded by Neutron diffraction.

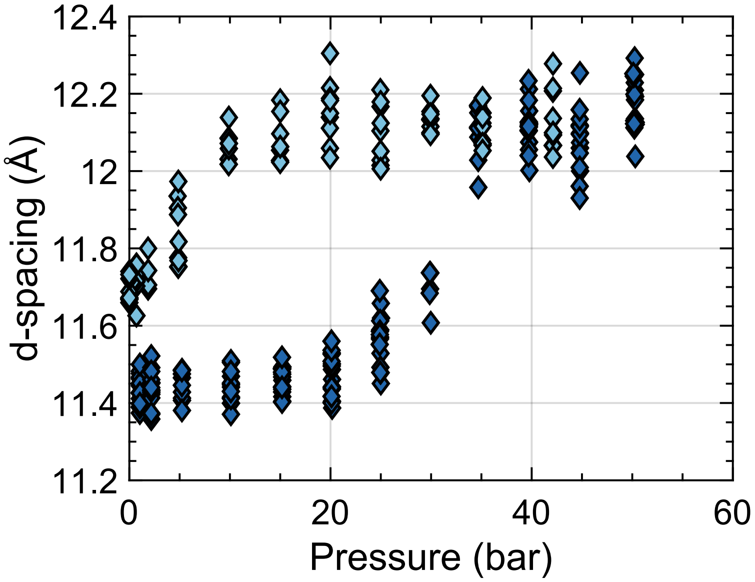

Figure S6: Evolution of the most prominent feature of the (002) Bragg reflection as a function of pressure at 26 °C for Ni-Hec$_{0.7}$ recorded by Neutron diffraction.

Additional adsorption measurements

A N_2 physisorption measurement was recorded for Ni-Hec$_{0.5}$ on a Quantachrome Autosorb 1 at 77 K. The sample was degassed overnight at 150 °C prior to the measurement. The results are presented in Figure S7, and by BET analysis a surface area of 1.8 m^2/g was calculated.

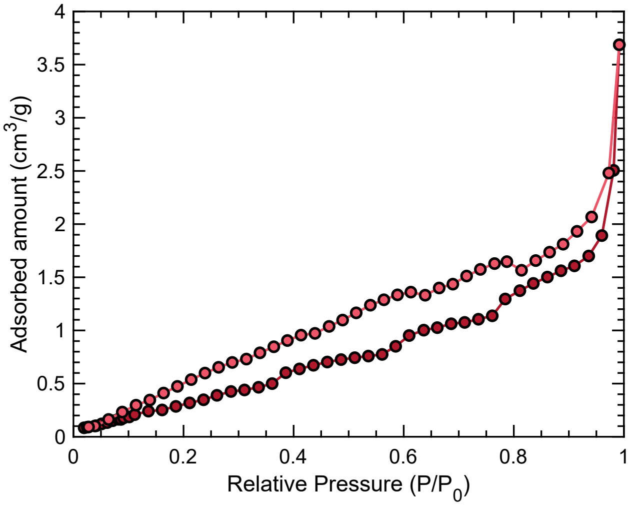

Figure S7: N_2 physisorption isotherm of Ni-Hec$_{0.5}$.

Table S1: Densities obtained from He-isotherms.

Sample	Ni-Hec$_{0.3}$	Ni-Hec$_{0.5}$	Ni-Hec$_{0.7}$
Density (g/cm^3)	2.32	2.35	2.43

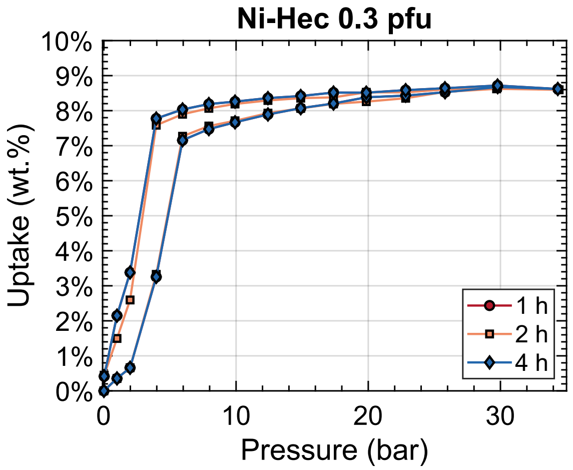

(a) Ni-Hec$_{0.3}$

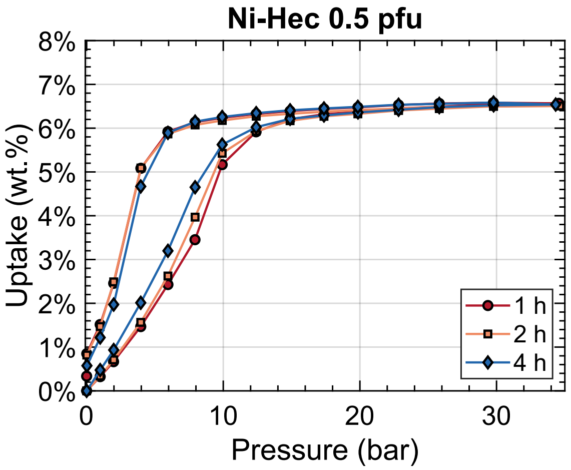

(b) Ni-Hec$_{0.5}$

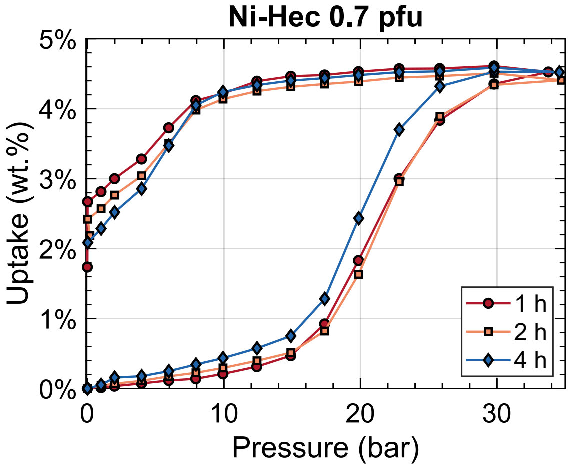

(c) Ni-Hec$_{0.7}$

Figure S8: Gravimetric adsorption measurements of Ni-Hec with layer charges a) Ni-Hec$_{0.3}$, b) Ni-Hec$_{0.5}$ and c) Ni-Hec$_{0.7}$ equilibrated at each pressure step for 1 h, 2 h and 4 h.

References

(S1) Hoffmann, J.-U.; Reehuis, M. E2: The Flat-Cone Diffractometer at BER II. *Journal of large-scale research facilities JLSRF* **2018**, *4*, 129.

(S2) Loch, P.; Hunvik, K. W. B.; Puchtler, F.; Weiß, S.; Seljelid, K. K.; Røren, P. M.; Rudic, S.; Raaen, S.; Knudsen, K. D.; Bordallo, H. N., et al. Spontaneous formation of an ordered interstratification upon Ni-exchange of Na-fluorohectorite. *Applied Clay Science* **2020**, *198*, 105831.

(S3) Hunvik, K. W. B.; Loch, P.; Cavalcanti, L. P.; Seljelid, K. K.; Røren, P. M.; Rudic, S.; Wallacher, D.; Kirch, A.; Knudsen, K. D.; Rodrigues Miranda, C., et al. CO2 Capture by Nickel Hydroxide Interstratified in the Nanolayered Space of a Synthetic Clay Mineral. *The Journal of Physical Chemistry C* **2020**, *124*, 26222–26231.
